# Successful treatment of an elderly patient with lung squamous cell carcinoma by tislelizumab and chemotherapy: a case report with novel imaging findings

**DOI:** 10.3389/fimmu.2025.1543114

**Published:** 2025-03-24

**Authors:** Lufan Xu, Xinxin Ma, Yang Yang, Zhiqiang Ding, Yi Luo

**Affiliations:** ^1^ Affiliated Hospital of Integrated Traditional Chinese and Western Medicine, Nanjing University of Chinese Medicine, Nanjing, China; ^2^ Nantong Tumor Hospital, Tumor Hospital Affiliated to Nantong University, Nantong, China

**Keywords:** lung squamous cell carcinoma, tislelizumab, PD-1 inhibitor, immunotherapy, chemotherapy

## Abstract

The advent of immunotherapy has transformed the therapeutic landscape for inoperable, locally advanced Non-Small cell lung cancer (NSCLC), particularly for lung squamous cell carcinoma (LUSC) with a predominance of negative driver genes. Based on the results of clinical trials such as KEYNOTE-042 and KEYNOTE-407, PD-1/PD-L1 inhibitors are now recognized as the standard of care for first-line or second-line treatment in many countries. Among the 17 immune checkpoint inhibitors sanctioned in China, tislelizumab, a domestically developed PD-1 inhibitor, enjoys broad application. Here, we present a case of a patient with LUSC who attained complete remission by cyst formation with the combination of tislelizumab and chemotherapy. Despite the absence of expression data for this patient, imaging studies revealed a reduction in the primary lesion size and the emergence of an uncommon cystic alteration post-treatment with sequential immunochemotherapy and tislelizumab monotherapy. As per the most recent follow-up, the lesion has vanished entirely. This outcome holds significant implications for the treatment of driver gene-negative LUSC by tislelizumab.

## 1 Introduction

Data from 2022 (1) reveal that lung cancer continues to hold the highest incidence and mortality rates globally. Non-Small cell lung cancer (NSCLC) constitutes the majority of lung cancer cases (2), among which 20-30% are lung squamous cell carcinoma (LUSC). Contrasted with lung adenocarcinoma (LUAD), in which driver mutations in the epidermal growth factor receptor (EGFR) and other genes are detectable with high sensitivity, LUSC is challenging to identify, resulting in limited intervention options (2012; 3). The identification of immune checkpoints like PD-1/PD-L1 and CTLA-4 has spurred the extensive application of immune checkpoint inhibitors (ICIs), pioneering new therapeutic pathways in oncology and enabling LUSC patients to reap the benefits of immunotherapy (4–6). The latest National Comprehensive Cancer Network (NCCN) guidelines advocate for anti-PD-1/L1 monotherapy as the standard second-line treatment for NSCLC, and PD-1 inhibitor monotherapy or its combination with chemotherapy as the standard first-line treatment for advanced NSCLC. For LUSC patients exhibiting PD-L1 positivity ≥50%, the recommended first-line treatment is immune monotherapy or a combination of chemotherapy and immunotherapy; for those with 1–49% PD-L1 positivity, the recommended first-line regimen is chemotherapy in conjunction with immunotherapy (7).

Immune checkpoint inhibitors, such as PD1/PD - L1, especially PD - L1 inhibitors, can concurrently bind to PD - L1 present on the surfaces of both tumor cells and antigen - presenting cells (APCs). This interaction restores T cell - mediated anti - tumor immunity (8). These inhibitors possess the capacity to impede the binding between PD - L1 on tumor cells and B7 - 1 on T cells (9, 10), thereby comprehensively activating T cells. Moreover, the inhibitors can also alleviate the self - inhibition of dendritic cells (11), reinforcing anti - tumor immunity from multiple perspectives.

Combining immunotherapy with chemotherapy can enhance the immune response through multiple mechanisms. It can induce immunogenic cell death (12), increase the exposure of tumor antigens, and promote antigen presentation and T cell activation (11). Chemotherapeutic agents can directly target and kill tumor cells, initiating apoptosis and subsequently releasing tumor antigens. Additionally, they can trigger the immunogenic cell death (ICD) pathway (13, 14). For instance, chemotherapeutic drugs like anthracyclines, platinum - based drugs, and taxanes can cause tumor cells to release damage - associated proteins during the process of cellular damage. These substances, known as damage - associated molecular patterns (DAMPs), include surface - exposed calreticulin (ecto - CRT), high - mobility group box 1 (HMGB1), heat shock proteins (HSPs), and extracellularly - released adenosine triphosphate (ATP). These DAMPs facilitate T cell infiltration and transform immunologically “cold” tumors into “hot” tumors. Ultimately, this leads to an augmentation of the tumor immune - inflammatory response and the induction of specific anti - tumor immune reactions.

In China, the standard treatment typically adheres to the most recent guidelines issued by CSCO(Chinese Society of Clinical Oncology) for the year of treatment. Regarding first-line treatment of LUSC lacking driver mutations, the Phase III IMpower110 study (15) demonstrated that atezolizumab markedly enhanced progression-free survival (PFS) and overall survival (OS) in patients with high PD-L1 expression in wild-type stage IV LUSC as compared to chemotherapy. The subsequent KEYNOTE-024 (16) and KEYNOTE-042 (17) studies corroborated that compared to chemotherapy, pembrolizumab significantly prolonged PFS and OS, diminished the frequency of adverse reactions, and substantially reduced the mortality risk for patients with PD-L1 TPS ≥ 1.Furthermore,the KEYNOTE-407 study revealed that the combination of immunotherapy and chemotherapy offered benefits to patients across various PD-L1 expression subgroups, as evidenced by the synergistic effects of pembrolizumab and chemotherapy. The RATIONALE-307 study indicated that the combination of tislelizumab with carboplatin and paclitaxel (or nab-paclitaxel) significantly extended PFS compared to the chemotherapy-only group (18).

Although the combination of immunotherapy and chemotherapy, as a first-line standard treatment for advanced LUSC was established in the KEYNOTE-407 study, treatment options for Chinese patients were previously constrained. Based on the data from the RATIONALE-307 study (18), drawing from a vast sample across various centers in China, tislelizumab received marketing approval, offering a more economical alternative to clinicians and cancer patients in China. This study presents a case of a patient with LUSC who achieved complete remission and cystic formation following treatment with the combination of tislelizumab and chemotherapy. Analogous cases have been documented in the literature. For instance, when inoperable tumors patients underwent 4-6 cycles of standard immunotherapy combined with chemotherapy some exhibited sustained regression of masses and lymph nodes and achieved complete remission, while others underwent surgical treatment after significant mass reduction and showed complete remission confirmed by pathology. The patient detailed in this report displayed an uncommon cystic radiological presentation after a mere 3 cycles treatment in combination with immunotherapy and chemotherapy. Subsequent to transitioning to the tislelizumab monotherapy due to adverse reactions associated with chemotherapy, successive follow-ups post 2 cycles revealed a progressive reduction in cyst size until it fully collapsed, culminating in complete remission. This distinctive radiological presentation visually underscores the synergistic advantage of combining immunotherapy with chemotherapy, which shows 1 + 1>2. It is noteworthy that the subsequent use of tislelizumab monotherapy was not less effective, potentially furnishing significant evidence for the use of tislelizumab as a monotherapy. Discussing of the issues encountered during the course of the combined immunotherapy-chemotherapy, including adverse reactions, benefits post-discontinuation, and immune efficacy assessment, is merited to augment clinical experience for LUSC patients treated with tislelizumab.

## 2 Case presentation

**Figure 1:**
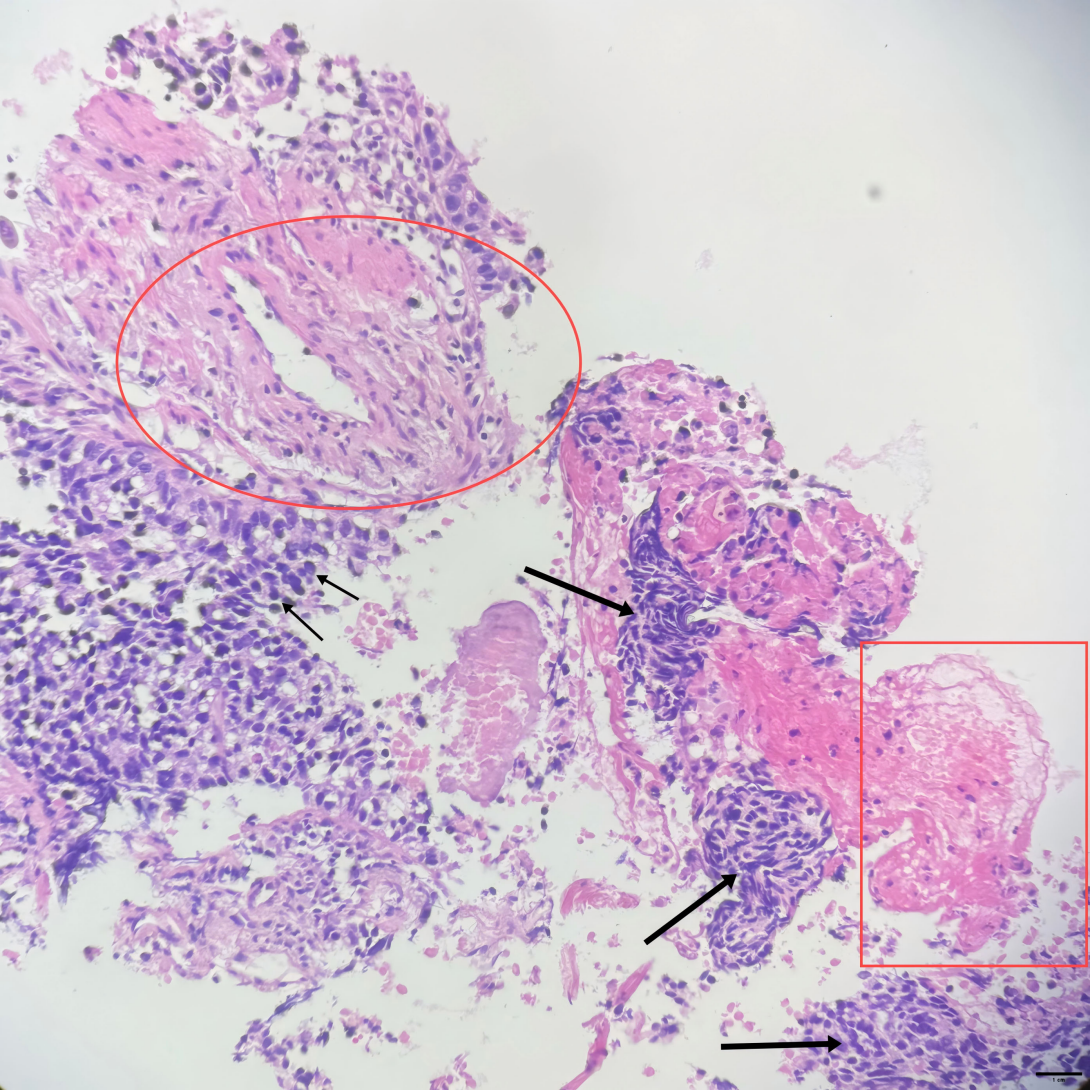
(10×ocular/20×objective lens, total magnification×200, scale bar = 100μm): Small arrow: Squamous cells with marked atypia demonstrating enlarged hyperchromatic nuclei, irregular nuclear contours, and increased nuclear-to-cytoplasmic ratio; Square: Intracellular keratinization (dyskeratosis); Large arrow: Tumor cell nests surrounded by stromal tissue; Circled area: Perivascular hyperplasia; Overall architecture: No normal lung tissue architecture is evident throughout the imageStaining.

A 73-year-old man presenting with paroxysmal cough and sputum accompanied by chest pain, weight loss, and exertional asthma was admitted to a local hospital on February 13, 2023. A chest computed tomography (CT) scan revealed a mass in the right upper lobe of the lung, raising the suspicion of a malignant tumor (MT). Subsequent positron electron tomography (PET)-CT indicated an irregular lobulated soft tissue mass in the apical segment of the right lung upper lobe, measuring approximately 4.1×3.5 cm and showing increased fluorodeoxyglucose (FDG) uptake (SUV value) and spiculated margins, suggestive of lung cancer (likely squamous cell carcinoma). There were tiny nodular shadows about 0.3 cm in diameter surrounding the mass, and the possibility of metastasis was not ruled out. Several enlarged lymph nodes were visible in regions 10R, 4R, and 2R, the largest measuring approximately 1.7 cm in diameter. that showed varying degrees of increased FDG uptake, suggesting possible metastasis in some lymph nodes. On March 3, 2023, a CT-guided biopsy of the right upper lobe lung mass was performed at our hospital. According to the pathology report (**Figure 1**), combined with the patient’s medical history and immunohistochemical markers, squamous cell carcinoma was considered (Note: CK7-, CK20-, CK5/6-, NapsinA-, CD56-, P40+, Syn-, CgA-, CD56-, TTF-1-, Ki-67 75%). As shown in the microscopic cellular morphology and tissue architecture of **Figure 1**, the entire field demonstrates complete loss of normal pulmonary tissue structure. Sheets of squamous cells with marked nuclear atypia are observed, accompanied by prominent keratinization features. PD-1/PD-L1 testing was not conducted. Based on the symptoms, signs, and test results, the patient was diagnosed with stage cT2bN2M0 right lung squamous cell carcinoma.

Due to old age, large lesions, and underlying diseases such as femoral head necrosis, surgery and radiotherapy are not considered for the patient. Following the guidelines of the Chinese Society of Clinical Oncology (CSCO),on March 14, 2023,immunotherapy combined with chemotherapy was started:paclitaxel liposome 240 mg on d1, carboplatin 500 mg on d1, and tislelizumab 200 mg on d2, every 3 weeks (q3w). Grade III thrombocytopenia occurred post-treatment, with a nadir of 30 × 10^9^/L. On April 18, 2023,the immunotherapy regimen was adjusted for the first cycle:tislelizumab 200 mg on d 0 + paclitaxel liposome 120 mg on d 1, 90 mg on d 8 + carboplatin 150 mg on d 2, 100 mg on d 3-5, every 3 weeks (q3w). However, on May 16, 2023, bacteremia and herpes zoster infection were observed, so antitumor treatment was halted. Vancomycin was given for anti - infection and anti - viral treatment. After active treatment, the first reexamination on May 29, 2023, with a repeat enhanced chest CT showed an irregular thin-walled cystic lesion in the right upper lobe with fine line compartments, measuring approximately 32×25×27 mm, with enlarged and moderately enhanced lymph nodes in the 10R, 4R, and 2R regions. Compared to the March 3, 2023 CT, the solid component of the right upper lobe mass had essentially disappeared, and the mediastinal lymph nodes were similar in size. Response assessment indicated partial remission, and the second cycle of immunotherapy combined with chemotherapy was administered on June 27, 2023.

After June 27, 2023, due to chemotherapy - induced bone marrow suppression and pre - existing heart and kidney dysfunction, the patient and family refused further chemotherapy. Anemia was corrected by infusion of leukocyte-reduced red blood cell suspension. Based on patient preferences, age, physical condition, imaging findings, and guidelines, two cycles of “tislelizumab 200 mg on day 0” monotherapy were administered on September 26 and November 27, 2023. Enhanced chest CT was again performed on November 27, 2023, showing an irregular cystic lesion approximately 31×20×22 mm beneath the pleura in the right upper lobe, with septation and moderately enlarged mediastinal lymph nodes. Compared to the previous scan, the cystic lesion in the right upper lobe had decreased slightly in size, with similar mediastinal lymph node enhancement. The patient and his family subsequently refused antitumor therapy due to personal reasons, and he was re-admitted for follow-up on April 8, 2024, after stopping immunotherapy for five months. This enhanced chest CT showed high-density cord-like shadows in place of the previously observed cystic lesions. Due to the lack of solid components of mediastinal window, it was impossible to evaluate the degree of enhancement of the dense cord-like shadows accurately, and the sizes of the mediastinal lymph nodes were similar to those seen previously.

**Figure 2:**
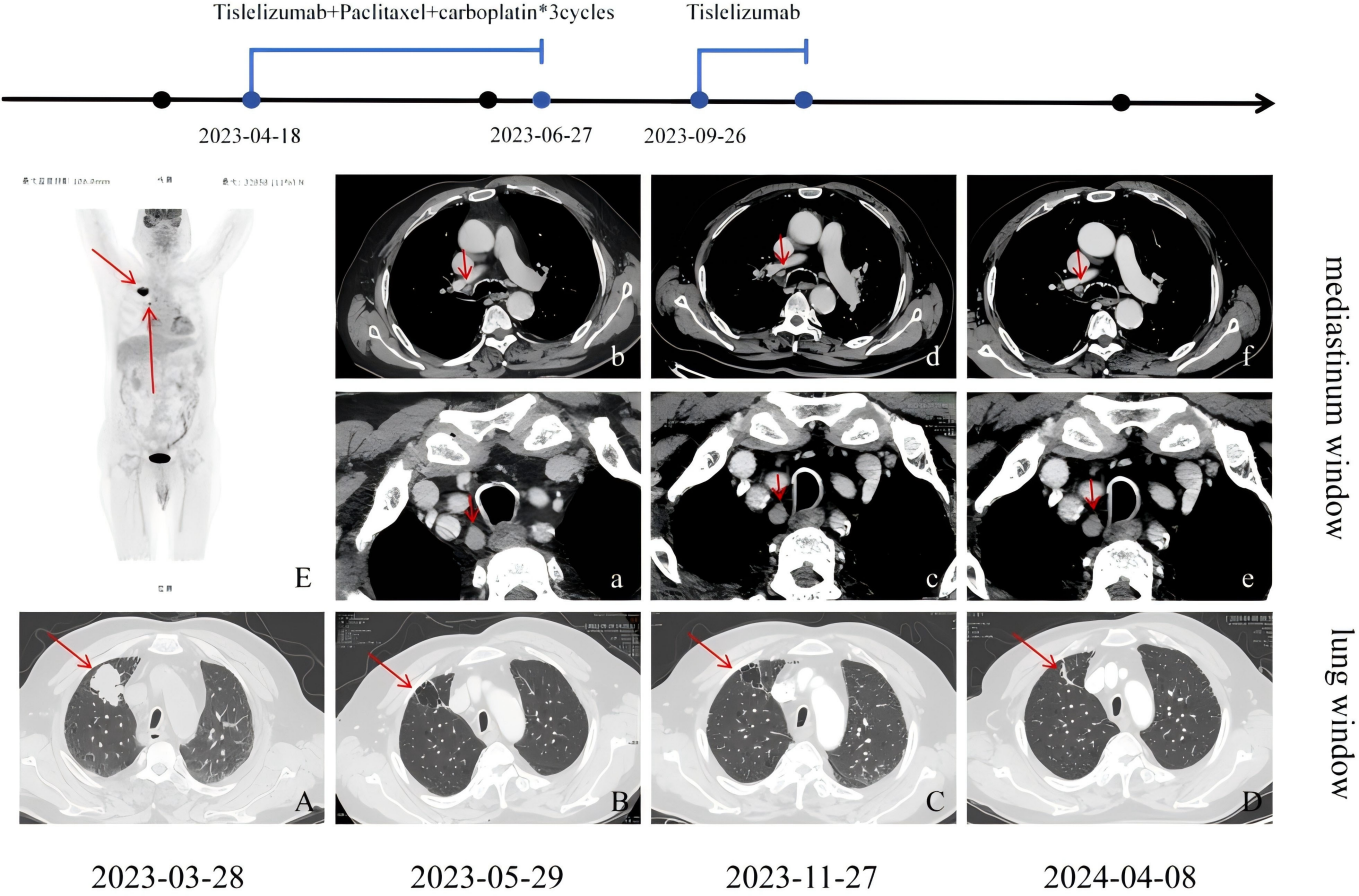
**(A)** Irregular soft tissue shadows are seen in the anterior segment of the upper lobe of the right lung, with lobulated and spiculated margins; **(B)** Following treatment for right upper lobe lung cancer, there was an irregular thin-walled cystic lesion measuring approximately 32×25×27 mm, with irregular linear septations and sparse lamellar dense shadows along the inner edge of the cyst; **(C)** The cyst was reduced in size compared to before, measuring approximately 31×20×22 mm, with almost complete collapse compared to before, presenting as cord-like dense shadows. **(E)** The FDG metabolism in the hilum of the right lung and mediastinal lymph nodes was increased to varying degrees, with the largest node having a diameter of approximately 17 mm. In the mediastinum, nodes in regions 10R **(b)**, 4R, and 2R **(a)** were enlarged with moderate enhancement, with the largest nodes in 10R and 2R measuring approximately 16×10 mm. Regions 10R **(d)**, 4R, and 2R **(c)** also showed several enlarged nodes with moderate enhancement, similar in size to previous findings. Regions 10R **(f)**, 4R, and 2R **(e)** showed several enlarged nodes with moderate enhancement, similar in size to previous findings.

**Figure 3:**
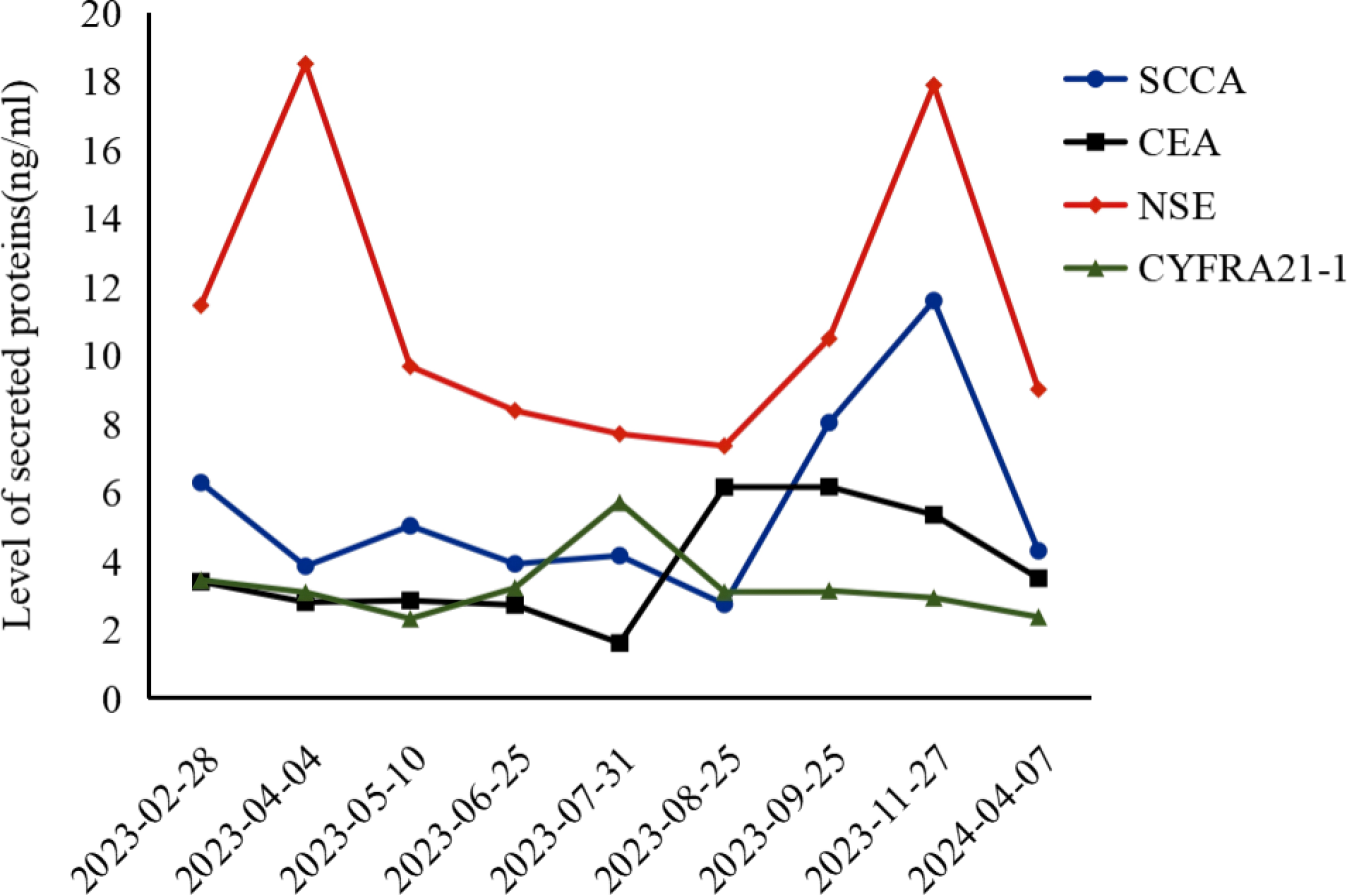
CSSA, Carcinoembryonic Antigen-related Sialic Acid-binding Adhesion Molecule; CEA, Carcinoembryonic Antigen; NSE, Neuron-Specific Enolase; CYFR21-1, Cytokeratin 19 Fragment.

To evaluate the curative effect more accurately, cranial MRI was performed, showing no metastases. Due to the patient’s and family members’ refusal to undergo PET-CT for the re-examination of metastatic lymph nodes and percutaneous pathological biopsy, we are only able to make a comprehensive assessment based on enhanced computed tomography (CT), tumor markers, and clinical symptoms. **Figure 2** clearly illustrates the patient’s imaging examination results spanning from 2023-03-28 to 2024-04-08, as well as the treatment regimens at different time intervals. The most recent CT reveals that the cystic cavity of the target lesion has completely collapsed, and the longest and shortest diameters of the mediastinal lymph nodes measure 9 mm. Concurrently, the levels of the serum tumor markers CSSA, CEA, NSE, and CYFR21-1 had decreased significantly compared to those observed on November 27, 2023,and the specific values are shown in **Figure 3**. According to the iRecist criteria, the efficacy was evaluated as complete remission.

## 3 Discussion

To date, there have been very few cases of complete remission achieved through drug treatment. A review of the literature identified a case report describing a patient with stage IVB LUSC who received a combination treatment of cisplatin/carboplatin, nab-paclitaxel, and tislelizumab. After 4 cycles, the patient showed a good response and achieved partial remission. Notably, the CT results indicated the disappearance of the main primary lesion in the left upper lung, resulting in the formation of a thick-walled cavity, while the lesion in the right lung was significantly reduced in size (CR1, **Table 1**). Additionally, there are related case reports where tislelizumab combined with chemotherapy was used for neoadjuvant therapy, resulting in successful reduction of the lesion, followed by surgery and subsequent complete pathological remission (CR2 and CR3, **Table 1**). Therefore, achieving complete remission through drug treatment alone in the present case is quite surprising and holds a certain significance and reference value in the real world.

**Table 1:** Case reports on the use of tislelizumab combined with chemotherapy for LUSC.

Case Id	Basic Information	Diagnosis	Driver Gene	PD-1 and PD-L1 Expression	Treatment Objective	Drugs	Surgery	Efficacy Evaluation	Treatment Plan After First Evaluation	Adverse Reactions	References
CR1	69-year-old male	Left lung IVB (cT4N2M1c) and right lung IVB (cT2N2M1c) LUSC	Not provided	Negative	First-line treatment	TP + Tislelizumab for 3 cycles, TC + Tislelizumab for 1 cycle	None	Partial remission	Tislelizumab	Hypothyroidism	(27)
CR2	45-year-old male	Inoperable stage IIIC (CT3N3MO) LUSC	Not provided	Not provided	Neoadjuvant treatment	TL + Tislelizumab for 2 cycles	Thoracoscopic left lower lung tumor resection and radical mediastinal lymph node dissection	Postoperative pathological complete remission	TL + Tislelizumab for 2 cycles, Tislelizumab for 12 months	Mild thyroid dysfunction	(28)
CR3	64-year-old male	Stage IIIB (cT3N2M0) right lung LUSC	No mutations	Negative	Neoadjuvant treatment	TC + Tislelizumab for 2 cycles	Thoracoscopic lung cancer resection	Postoperative pathological complete remission	Tislelizumab	Grade 2 bone marrow suppression	(29)

CR1-3: Case Report 1-3 TP: Paclitaxel + Cisplatin TC: Paclitaxel + Carboplatin TL: Nab-paclitaxel + LobaplatinPR: Partial response pCR: Pathological complete response.

In this case, the patient underwent re-examination CT after receiving two cycles of a standard regimen of immunotherapy combined with chemotherapy, and it was found that a cyst had formed at the original tumor site. This imaging presentation differs from what is commonly seen after lung cancer treatment. In this case, the solid component of the soft tissue mass in the right upper lobe disappeared rapidly after treatment, leaving irregular cysts. Since the cystic cavity shadow basically disappeared after regression, the presence of pre-existing cystic cavity changes in the lung, such as pulmonary bullae, pulmonary cysts and bronchiectasis, is not considered. In the absence of pathology, we believe that the formation of cystic cavities is caused by the following factors:(1) The tumor involved the small bronchi, causing local bronchial stenosis or obstruction, forming an “air valve effect,” resulting in air trapping (19); (2) The tumor had invaded the alveoli and the surrounding interstitial structure (20), causing alveolar fusion (21) and the formation of large cysts. When treatment was effective, after the tumor tissue disappeared, the remaining destroyed lung tissue formed irregular cysts, which then slowly collapsed. These cysts shrank gradually during the follow-up period, and re-examination after about one year showed that the cysts had collapsed, leading to the presentation of cord-like dense shadows. The mechanism of cystic cavities resulting from alveolar injury and pulmonary small airway obstruction may be similar to that of cystic lung cancer.

The formation of cystic cavities also has an impact on the direction of subsequent clinical decision-making. The chest enhanced CT on May 29 showed changes in the nature of the lesion, suggesting the disappearance of the tumor cells and the formation of cysts. However, previous PET-CT reports had suggested possible metastasis to surrounding lymph nodes, and the enhanced CT on May 29 still showed several enlarged, moderately enhanced lymph nodes in the mediastinum (**Figure 2**). At this time, the focus of the patient’s treatment lies in rapidly shrinking the tumor and controlling the disease progression. Therefore, continuing the immunotherapy combination treatment becomes the primary strategy after the first assessment.

Despite the promising efficacy of the combined immunotherapy and chemotherapy, the patient experienced grade III thrombocytopenia (with a nadir of 30×10^9^/L) and grade IV hemoglobin reduction (with a nadir of 49 g/L) on multiple occasions during the treatment. These were both considered to be chemotherapy - related bone marrow suppression. Moreover, severe bacteremia occurred, which significantly decreased the patient’s quality of life and led to a marked decline in the Karnofsky Performance Status (KPS) score. Meanwhile, the patient’s cardiac and renal functions remained suboptimal, with elevated levels of transaminase, creatinine, and B - type natriuretic peptide (BNP) to varying degrees. Based on these circumstances, the treatment team had thorough communication with the patient and their family to develop an individualized treatment plan suitable for this patient. On one hand, since the PD - L1 test had not been conducted, the PD - L1 expression was unclear, and it remained uncertain whether tislelizumab monotherapy could effectively maintain the current positive treatment trend. On the other hand, the “chemotherapy - free mode”, a subtractive approach, might ensure the continuation of the patient’s treatment plan and improve their quality of life simultaneously. Through patient - centered individualized diagnosis and treatment, the focus of treatment was ultimately defined as controlling the disease with low toxicity to ensure the quality of life. This approach achieved results that satisfied both the medical team and the patient.

ICIs primarily counteract immune suppression through specific inhibition of immune checkpoints, thereby enhancing existing antitumor immune responses at different stages of the tumor immune cycle. However, whether these responses persist after stopping immunotherapy is currently uncertain. The CheckMate 069 study showed that in some patients, treatment response can continue for a period even after discontinuation due to toxic reactions. It was found that 66% of patients continued to benefit after stopping immunotherapy, with complete response rates of 20%, partial response rates of 46%, and stable disease in 17%. The 2-year survival and PFS rates for cancer patients who discontinued immunotherapy were found to be 71% and 52%, respectively (22, 23). In this case, the patient discontinued treatment due to personal reasons after the last dose, and the discontinuation lasted for 4 months. Although the skin rash remained during this period, the first follow-up imaging showed complete disappearance of the target lesions, with the largest short-axis diameter of enlarged lymph nodes measuring 9 mm, while non-target nodules remained unchanged at 4 mm, demonstrating a favorable immune smearing effect. Follow-up after discontinuation of immunotherapy is particularly important for this patient, and the absence of PET-CT and percutaneous pathological biopsy poses certain risks for tumor recurrence and metastasis. We will conduct regular enhanced CT examinations to closely monitor the size and enhancement pattern of the lymph node region. By fully combining the patient’s clinical symptoms with laboratory test results, we aim to ensure the detection of the disease condition and timely updating of the treatment plan.

In a clinical trial comparing tislelizumab plus paclitaxel and cisplatin versus chemotherapy alone in phase IIIb to IV NSCLC patients, the levels of serum biomarkers, the overall response rate, and the disease control rate were all significantly better in the tislelizumab group, although the incidence of adverse reactions did not differ significantly (18). Among ICI-induced immune-related adverse events (24), 30-50% of patients developed toxic reactions of the skin, including rash, pruritus, psoriatic lesions, and lichenoid dermatitis. Case reports have suggested that while tislelizumab has good safety, it can also cause skin rash and potentially induce bullous pemphigoid (BP), a severe autoimmune disorder (25, 26).In this patient case, obvious rashes persisted during the immunotherapy - chemotherapy combination treatment and the subsequent monotherapy. Considering the patient’s history of femoral head necrosis, glucocorticoid treatment was not administered. During this period, timely consultations with dermatologists were carried out, and urea ointment was prescribed for treatment.

In addition to the rash, the patient manifested significant thrombocytopenia after the first treatment. Regarding this phenomenon, the local treatment team conducted an assessment of the bleeding risk. The patient displayed no obvious cutaneous ecchymoses or petechiae, had no epistaxis or oropharyngeal hemorrhage, and was given subcutaneous injection of rhTPO. In the efficacy evaluation of immunotherapy, about 80% of patients achieve their first remission around the first evaluation (6 weeks) or the second evaluation (12 weeks). After receiving 2 cycles of treatment, the evaluation of immunotherapeutic efficacy was of particular importance. However, at this time, the patient developed a high fever. Catheter blood culture revealed an infection of Acinetobacter baumannii, and peripheral blood culture demonstrated an infection of Pantoea agglomerans. After the treatment team administered piperacillin - tazobactam for catheter sealing, the C - reactive protein (CRP) level exhibited a decrease. Later, the patient had a high fever again. Re - examination of catheter and peripheral blood cultures indicated infections of Staphylococcus haemolyticus and Staphylococcus epidermidis. Taking into comprehensive consideration the concurrent bacteremia, the antibiotic was adjusted to vancomycin for anti - infection treatment. After treatment, the patient’s symptoms improved significantly. Re - examination of whole blood and catheter - derived blood cultures showed no bacterial growth for five consecutive days. Meanwhile, numerous erythemas and clustered blisters appeared on the left axilla and the flexor side of the upper arm of the patient. Herpes zoster was suspected, and symptomatic treatments such as antiviral therapy and nerve - nourishing treatment were given, and the patient’s renal function was closely monitored. Thanks to the good control of complications, the patient underwent the first evaluation as scheduled on May 29, 2023, and obtained gratifying results, providing sufficient confidence and basis for subsequent continuous treatment. The timely management of adverse reactions together with adjustments in treatment guided by clinical and guideline recommendations proved beneficial overall for this patient.

Currently, there are few reported cases of elderly patients with lung squamous cell carcinoma being successfully treated by PD-1 inhibitors combined with chemotherapy and subsequently forming cavities. This case is limited by the small amount of puncture samples, resulting in an insufficient number of subsequently prepared tissue white slides, and thus the PD-L1 status cannot be determined. However, after the sequential application of chemotherapy combined with immunotherapy and single-agent immunotherapy, a favorable outcome has still been achieved. Although this case still has certain limitations, it nevertheless provides a reference for the timing of clinical immunotherapy application and how to proceed with treatment in the specific situation of cyst formation.

## Data Availability

The original contributions presented in the study are included in the article/supplementary material. Further inquiries can be directed to the corresponding authors.
